# Age patterns of severe paediatric malaria and their relationship to *Plasmodium falciparum *transmission intensity

**DOI:** 10.1186/1475-2875-8-4

**Published:** 2009-01-07

**Authors:** Emelda A Okiro, Abdullah Al-Taiar, Hugh Reyburn, Richard Idro, James A Berkley, Robert W Snow

**Affiliations:** 1 Malaria Public Health and Epidemiology Group, Centre for Geographic Medicine, KEMRI-Wellcome Trust Collaborative Programme, Kenyatta National Hospital Grounds P.O. Box 43640-00100, Nairobi, Kenya; 2 Faculty of Medicine and Health Sciences, Sana'a University, P.O. Box 13078, Sana'a, Yemen; 3 London School of Hygiene and Tropical Medicine, Keppel St, London, WC1E 7HT, UK; 4 Kilimanjaro Christian Medical Centre P.O. Box 2228 Moshi, Tanzania; 5 Department of Paediatrics, Mulago Hospital/Makerere University Medical School Kampala, Uganda; 6 Kenya Medical Research Institute, Centre for Geographic Medicine Research – Coast, Kilifi, Kenya P.O. Box 230-80108, Kilifi, Kenya; 7 Centre for Tropical Medicine, Nuffield Department of Clinical Medicine, University of Oxford, CCVTM, Oxford, OX3 9DS, UK

## Abstract

**Background:**

The understanding of the epidemiology of severe malaria in African children remains incomplete across the spectrum of *Plasmodium falciparum *transmission intensities through which communities might expect to transition, as intervention coverage expands.

**Methods:**

Paediatric admission data were assembled from 13 hospitals serving 17 communities between 1990 and 2007. Estimates of *Plasmodium falciparum *transmission intensity in these communities were assembled to be spatially and temporally congruent to the clinical admission data. The analysis focused on the relationships between community derived parasite prevalence and the age and clinical presentation of paediatric malaria in children aged 0–9 years admitted to hospital.

**Results:**

As transmission intensity declined a greater proportion of malaria admissions were in older children. There was a strong linear relationship between increasing transmission intensity and the proportion of paediatric malaria admissions that were infants (R^2 ^= 0.73, p < 0.001). Cerebral malaria was reported among 4% and severe malaria anaemia among 17% of all malaria admissions. At higher transmission intensity cerebral malaria was a less common presentation compared to lower transmission sites. There was no obvious relationship between the proportions of children with severe malaria anaemia and transmission intensity.

**Conclusion:**

As the intensity of malaria transmission declines in Africa through the scaling up of insecticide-treated nets and other vector control measures a focus of disease prevention among very young children becomes less appropriate. The understanding of the relationship between parasite exposure and patterns of disease risk should be used to adapt malaria control strategies in different epidemiological settings.

## Background

During the 1980's and 1990's a series of epidemiological observations were reported on the age and clinical patterns of severe malaria in African children across a range of *Plasmodium falciparum *transmission intensities [1-4]. It appeared from these early observations that the intensity of transmission affected the mean age and clinical features of severe disease and rates of disease showed a nonlinear relationship with transmission intensity, stimulating much heated debate and commentary [5-9].

Subsequent to these earlier studies there has been a renaissance in the clinical epidemiology of severe paediatric malaria across a wide range of different transmission settings, leading to descriptions of severe paediatric malaria from Sudan [10], Mozambique [11], Tanzania [12,13], Mali [14], Niger [15], Kenya [16], Uganda [17,18], Yemen [19], Ghana [20,21] and Zambia [22]. In addition, there have been several further attempts to compare the epidemiological patterns of severe malaria between sites of different transmission intensity in Gabon [23], Burkina Faso [24], Uganda [17], Sudan [25], Tanzania versus Mozambique [26], and one study that compared severe malaria risks at different altitudinal transmission limits in Tanzania [13]. The consensus view of all studies is that as the intensity of *P. falciparum *transmission increases, the mean age of severe malaria decreases. Less consistent is the reported relationship between clinical syndromes of severe paediatric malaria and transmission intensity.

There are three limitations of many of the reported ecological comparisons. First, they often compare data from very few sites, reducing the contextual ranges of the observations and thus their wider validity. Second, the measures of transmission intensity used in most studies are often imperfect or assumed rather than measured [13,17,23,25]; or not matched to the period of clinical surveillance under review [11,26]. Finally, despite an increased number of observational studies of severe malaria, they often do not cover the same age range, nor do they use similar diagnosis and surveillance methods.

To circumvent some of these limitations of cross-site comparisons and increase the power to generalize from observations, data from 17 sites across seven countries are presented. The standardization of metrics used to define transmission intensity in each site have significantly improved, have been temporally matched to the clinical surveillance period and attempts have been made to ensure standardization in surveillance and diagnostic methods between survey observations, to examine the relationship between age patterns of hospitalized paediatric malaria and *P. falciparum *transmission intensity.

## Methods

### Clinical surveillance

In this review we have assembled clinical admission data from 17 communities served by 13 hospitals. The surveillance sites were selected on the basis of having established clinical and parasitological surveillance, often to specifically study the pathophysiology, management and epidemiology of severe paediatric malaria.

### Study sites

**Figure 1:**
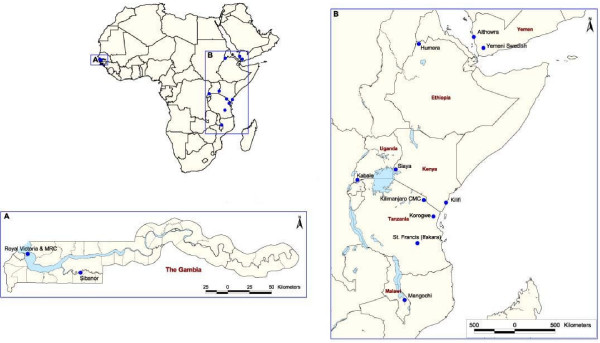
**Hospital sites included in the study of the age and clinical epidemiology of hospitalized paediatric malaria**: Kilifi District Hospital (A), Althowra Hospital (B), Royal Victoria Hospital & the Medical Research Council hospital (C), Yemeni Swedish Hospital (D), Kilimanjaro CMC (E), Humera district Hospital (F), Kabale regional refferal Hospital (G), Mangochi district Hospital (H), Sibanor Clinic (I), Korogwe District Hospital (J), Siaya District Hospital (K), St Francis Hospital (L).

The 17 communities represent a wide range of malaria ecology typical of the *P. falciparum *endemic world (Figure 1, Table 1). These include six sites where the clinical pattern of severe malaria and its relationship to transmission intensity were described by Snow and colleagues in the early 1990s [2,3]; three communities in Kenya (Kilifi North, Kilifi South and Siaya), two in The Gambia (Bakau and Sukuta) and one community in Tanzania (Ifakara). The data collected in Ifakara [2] were re-assembled to ensure that only clinical admissions from Namawala and Michenga villages were included to spatially correspond directly to measures of malaria transmission. Three additional temporally discrete surveillance periods have been included among approximately similar communities studied during the 1990's around Kilifi District Hospital, on the Kenyan Coast including Kilifi North, Chonyi and Junju investigated approximately ten years after clinical descriptions in these approximately matched areas. Data from a further six sites in Africa, where identical surveillance methods were reported, were also included: Humera, Ethiopia [27], Foni Kansala, The Gambia (reported in [28]), Mponda, Malawi (reported in [28]), Magunga and Kilimanjaro, Tanzania [13] and Kabale, Uganda [17]. Two additional sites in the Arab peninsular make up the last study sites and are regarded here as sharing a similar malaria and dominant vector species ecology to the horn of Africa; these include clinical admission data from the Yemeni-Swedish Hospital, in Taiz, and Althowra Hospital in Hodeidah City [19].

**Table 1:** Description of clinical surveillance sites and the characteristics of the catchment populations in relation to transmission intensity (*Pf*PR_2–10_-*Plasmodium falciparum *parasite prevalence in children 2 to 10 years).

**Study Site** **[Map Reference]**	**Dates** **(years)**	**Malaria admissions**	**BCS^1 ^≤ 2 recorded** **(Y/N)**	**SMA^2 ^recorded** **(Y/N)**	***Pf*PR _2–10_** **(years recorded) [number examined]**
Kilifi North, Kenya [A]	2004–07 (4)	712	Y	Y	1.3 (2005–07) [828]
Hodeidah, Yemen [B]	2002–04 (1.75)	283	Y	Y	1.7 (2005–06) [5886]
Bakau, The Gambia [C]	1992–94 (3)	99	Y	N	2.1 (1988) [386]^3^
Taiz, Yemen [D]	2002–04 (1.75)	1049	Y	Y	5.7 (2005–06) [4908]
Kilimanjaro, Tanzania [E]	2002–03 (1)	162	Y	Y	6.2 (2001–02) [382]
Humera, Ethiopia [F]	1994–95 (1)	458	N	Y	12.6 (1995) [616]
Kabale, Uganda [G]	2002–03 (1.5)	160	Y^5^	Y	18.0 (2006) [64]^4^
Kilifi South Junju, Kenya [A]	2005–07 (3)	92	Y	Y	25.9 (2005–07) [1601]
Mponda, Malawi [H]	1994–95 (1)	356	Y	Y	33.0 (1996)
Foni Kansala, The Gambia [I]	1994–95 (2)	193	Y	Y	34.1 (1991–92) [117]
Korogwe, Tanzania [J]	2002–03 (1)	3948	Y	Y	34.9 (2000–02) [927]
Sukuta, The Gambia [C]	1992–95 (4)	605	Y	N	42.4 (1996) [125]
Kilifi South Chonyi, Kenya [A]	1999–01 (3)	346	N	Y	43.0 (1999–01) [1918]
Kilifi North, Kenya [A]	1990–95 (5)	1358	Y	Y	51.9 (1995) [540]
Siaya, Kenya [K]	1992–96 (3)	715	Y	Y	75.1 (1995) [570]
Kilifi South, Kenya [A]	1992–96 (4)	766	Y	Y	76.9 (1996) [212]
Namawala/Michenga, Tanzania [L]	1991–92 (1)	144	Y	Y	87.5 (1989–91) [3947]

^1^BCS – Blantyre Coma Score

^2 ^SMA Severe Malaria Anaemia defined as Hb <5 gm/dl or PCV<15%

^3 ^The estimate of *Pf*PR was not temporally matched however it was regarded as a legitimate estimate for this peri-urban community four years later when the clinical surveillance data began.

^4 ^Kabale is a high altitude area and while there were 3 years difference in the estimation of *Pf*PR and the clinical surveillance period the estimate of infection prevalence is regarded as a good approximation.

^5 ^The investigators used a BCS ≤ 2 to describe cerebral malaria in children aged less than 5 years old and a Glasgow Coma score [32] of ≤ 8 for children 5–9 years.

### Surveillance and diagnosis

At each hospital all paediatric admissions routinely undergo a screening procedure where symptom histories are recorded. the definition of paediatric is restricted to children aged less than ten years. Each child was examined on admission and a blood sample taken for malaria parasitology and haematology. Diagnosis was supported by detailed clinical examination; all clinical and laboratory data are reviewed by investigating physicians who established a primary diagnosis (defined as the principal reason for the child's admission). A primary diagnosis of malaria was made when a child had a positive blood smear and no other detectable cause for the clinical presentation, after a review of all available clinical and haematological data and where indicated, X-ray and microbiological data. Two clinically important complications of severe malaria in African children are cerebral malaria and severe malarial anaemia [29,30]. Consciousness on admission is recorded at most sites according to the Blantyre Coma Score (BCS) based on verbal, motor, and gaze responses to stimulation [29,31]. To allow comparison across studies a BCS of 0, 1, or 2 (4 or 5 considered normal depending on age [29]) was used to define coma and cerebral malaria in the presence of malaria infection and absence of other causes for the clinical presentation. In Kabale, a BCS of ≤ 2 was used to describe cerebral malaria in children aged less than 5 years old and a Glasgow Coma score [32] of ≤ 8 for children 5–9 years Severe malaria anaemia was defined as a diagnosis of malaria with an admission haemoglobin level of less than 5.0 g/dl or PCV of less than 15%.

### *Plasmodium falciparum *transmission intensity

Cross-sectional estimates of *P. falciparum *infection prevalence from the communities served by the study hospital sites were assembled from information available as part of The Malaria Atlas Project [33] database [34,35]. Infection prevalence estimates were identified where they were spatially congruent, within 20 km of the hospital (Authors, unpublished data), and temporally congruent, during the years of the hospital surveillance. This restriction increases the accuracy of assigning transmission intensity to each of the clinical admission series. For each series of parasite prevalence survey data, the age-ranges reported varied between surveys and these were standardized to a single age range 2–10 years (*Pf*PR_2–10_) using algorithms described elsewhere [36].

## Results

The study series included a total of 11,446 children admitted to the 13 hospitals with a primary diagnosis of malaria confirmed by microscopy from the 17 communities covering a total of 17 survey years between 1990 and 2007 (Table 1, Figure 1). The communities served by the hospitals represented the entire range of transmission intensity from *Pf*PR_2–10 _values of 1% in Kilifi North between 2004–07 to as high as 87% in Namawala/Michenga villages in Tanzania in the early 1990s (Table 1).

**Figure 2:**
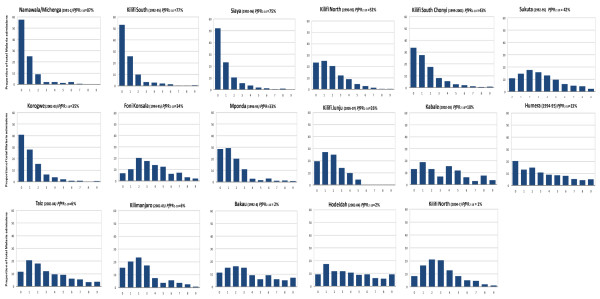
**Age distribution of hospitalized malaria from 17 communities arranged by decreasing *Pf*PR_2–10 _(*Plasmodium falciparum *parasite prevalence in children 2 to 10 years)**. The bars denote the percentage of children in each single age group of all malaria admissions 0–9 years at each site.

To examine the corresponding age-patterns of malaria admission against transmission intensity we computed the proportion of all malaria admissions by single years of age 0 to 9 years at each site, arranged in descending order of *Pf*PR_2–10 _in Figure 2. Across the 17 communities the tendency was toward a greater proportion of older children presenting as *Pf*PR_2–10 _estimates decreased and a greater proportion of younger children admitted where *Pf*PR_2–10 _was higher. It was nevertheless notable, that even at very low estimates of *Pf*PR_2–10_, the proportion of cases after the fourth birthday was lower than in early childhood (last five panels in Figure 2).

**Figure 3:**
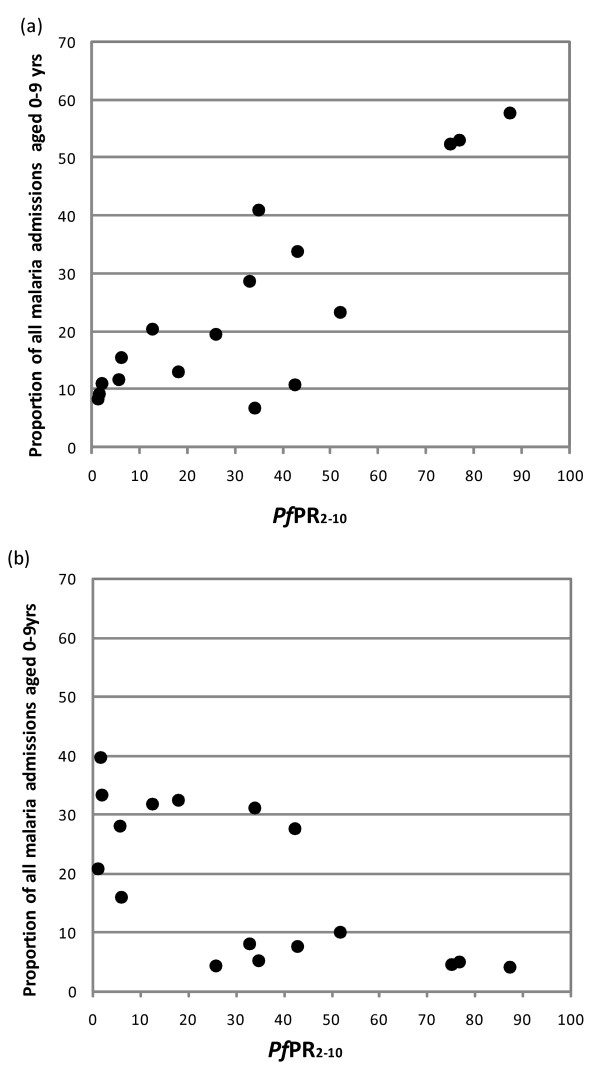
**Age specific proportion of total hospitalized paediatic malaria cases under different transmission intensities (x-axis; *Pf*PR_2–10_-*Plasmodium falciparum *parasite prevalence in children 2 to 10 years)**. The graphs show for each study sites the proportion of total malaria cases in children < 1 year (Figure 3a) and the proportion of total malaria cases in children 5–9 years (Figure 3b).

For a number of reasons, that are expanded on in the discussion, the public health and intervention significance of clinical risks in infancy is of programmatic importance. Among the six communities where the *Pf*PR_2–10 _estimate was ≥ 40%; approximately 40% of all malaria admissions were in children aged < 1 year. This compared with an average of 20% of all malaria admissions in infancy among the eight sites with a *Pf*PR_2–10 _between 5 and 39%, and 10% of all admissions at the three sites where *Pf*PR_2–10 _was recorded as < 5%. There is a direct and strong linear relationship between increasing *Pf*PR_2–10 _and the proportion of paediatric malaria admissions that are infants (R^2 ^= 0.73, p < 0.001; Figure 3a) and the converse relationship with the proportion of admissions that are aged between 5–9 years (R^2 ^= 0.47, p = 0.002; Figure 3b). A few outliers are worth identifying: first Junju, Kilifi South, Kenya with a *Pf*PR_2–10 _estimate of 26% had no admissions above 5 years of age during the observation period; second, at Kilifi North between 2004–07 *Pf*PR_2–10 _was recorded as 1% and Kilimanjaro with a *Pf*PR_2–10 _estimate of 6%, however, both had considerably more children admitted aged 0–4 years compared to those 5–9 years of age. Finally, the Kilifi South (Chonyi) admission series documented between 1999 and 2001 shows a high proportion of infants while transmission intensity is intermediary between two sites where the infant admissions are much lower.

The observations summarized in Figure 2 include overlapping communities seen at different times with very different estimates of *Pf*PR_2–10 _during each observation period: Kilifi North in the 1990s and 2000s and two closely located communities surveyed between 1999 and 2007 south of Kilifi District Hospital and corresponding to Kilifi South surveyed in 1990's. In the same communities over the two time periods transmission had dropped dramatically. In Kilifi North over ten years *Pf*PR_2–10 _dropped from 52% to 1% and at Kilifi South (1992–1995) and the nested area of Chonyi (1999–2001) corresponding *Pf*PR_2–10 _estimates were 77% and 43% respectively. In both areas the age patterns of malaria admissions had shifted toward older children as *Pf*PR_2–10 _declined but the most dramatic age-shift was observed at Kilifi North with the largest decline in *Pf*PR_2–10 _over a longer time frame.

**Figure 4:**
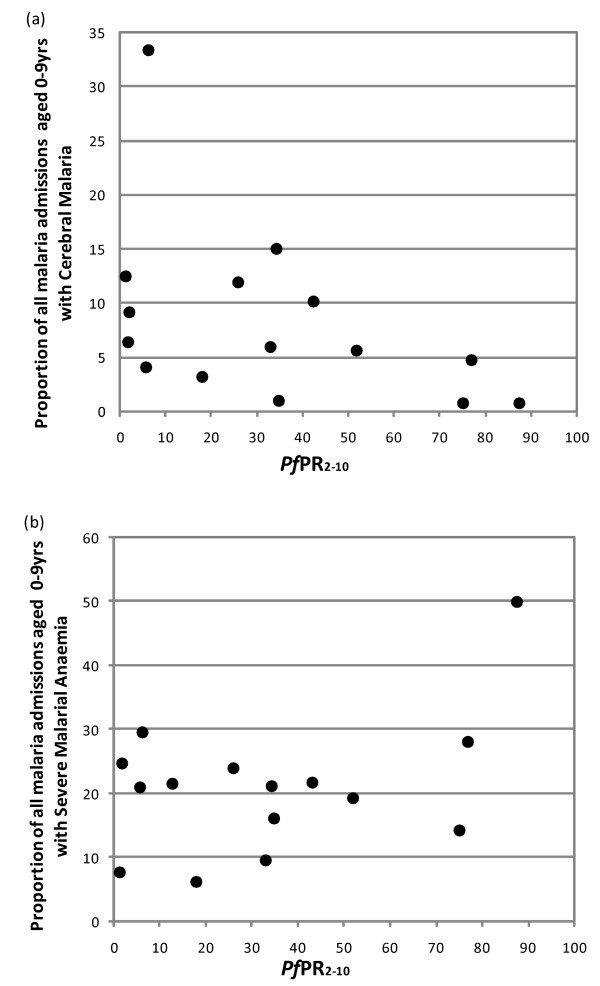
**Proportion of total malarial cases diagnosed with clinical syndrome of cerebral malaria (Figure 4a) and severe malarial anaemia (Figure 4b) under different transmission intensities (*Pf*PR_2–10 _– *Plasmodium falciparum *parasite prevalence in children 2 to 10 years)**.

The hospitals that routinely recorded unconsciousness using a BCS or anaemia on admission are shown in Table 1. Across this admission series of 10,642 and 10,742 cases of paediatric malaria respectively, cerebral malaria was reported among 4% and severe malaria anaemia among 17% of admissions. The relationship between the proportion of admissions presenting with cerebral malaria (BCS ≤ 2) and *Pf*PR_2–10 _is shown in Figure 4a. This suggests that at higher transmission intensity cerebral malaria is a less common presentation compared to lower estimates of *Pf*PR_2–10_, however, without the very high proportion of cerebral malaria cases reported at Kilimanjaro this relationship is less convincing. Similarly excluding the community of Nyamawala/Michenga in Tanzania where 50% of all admissions had a PCV <15% there appears to be no direct relationship between increasing transmission intensity and increase in proportion of severe malaria anaemia cases (Figure 4b).

## Discussion

There is mounting evidence that the epidemiology of malaria infection and disease risks are in transition is some parts of Africa, in part as a result of scaling of the provision of insecticide treated nets (ITN) and adoption of new effective therapeutics [37-41]. How changing the natural risks of parasite exposure by vector control will alter the clinical epidemiology of severe, complicated disease in young African children was the subject of concern over 50 years ago [42,43] and interest in this area re-emerged ten years ago following early comparisons of the clinical outcomes of infection in different transmission settings [3,4,8,44].

There are very few serial, long-term clinical observations of severe paediatric malaria in areas where transmission intensity is in transition [41]. Thus to understand the relationships between transmission intensity and disease outcome we must default to cross-sectional estimations of risk and exposure from different settings to infer what might happen if single communities transitioned between exposure states. Ecological comparative observational epidemiology is not without its limitations. An attempt has been made to standardize observations across 17 communities to minimize methodological measurement differences in the study of the clinical epidemiology of hospitalized paediatric malaria and transmission. Here *Pf*PR_2–10 _estimates of transmission contemporary with the clinical observations were used to ensure congruence between exposure and outcome.

One of the most striking observations was the relationship between increasing transmission intensity and the predominant age of paediatric malaria admissions (Figures 2, 3a and 3b). Among communities where *Pf*PR_2–10 _is ≥ 40% more than 40% of malaria admissions to paediatric wards were infants, compared to only 10% in areas where *Pf*PR_2–10 _is < 5%. These observations are consistent with the view that the speed of acquired clinical immunity scales with the frequency of parasite exposure since birth [3,4,45]. Interestingly from the sites where the intensity of transmission was very low (Figure 2), there remains evidence of some acquired functional immunity as expressed by the continued decline after the fourth birthday in the proportion of overall malaria admissions. This was most notable at Taiz (*Pf*PR_2–10 _6%) and Bakau (*Pf*PR_2–10 _2%). These declining risks with age under very low parasite exposure from birth suggest that only a few parasite exposures might confer a degree of clinical immunity [46] or age itself modifies risks of hospitalized malaria [47].

Despite the overall linear pattern of proportions of admissions aged less than one year (Figure 3a) or greater than five years (Figure 3b) with increasing transmission intensity there were some interesting exceptions to this general pattern. At two coincidentally matched sites investigated between ten years apart (Kilifi North) and five years apart (Kilifi South versus Chonyi) on the Kenyan coast the age-patterns were not as one might have anticipated based on the age patterns of disease seen in areas with very similar *Pf*PR_2–10 _(Figure 2). Both sites did show a changing age pattern of disease presentation with decreasing transmission intensity but had not resulted in an age pattern similar to those of historically similar transmission intensity in their second observation period. What these data might suggest is that the cross-sectional investigation of the clinical epidemiology of hospitalized malaria during a period of transmission transition, inevitably results in the study of older children exposed to different transmission intensity risks at different times in their young lives with a cohort effect of accumulated acquired immunity. Thus the "true" age pattern in a community would take some time to stabilize. This phenomenon might also explain the slightly divergent patterns seen at the two South Eastern sites in Tanzania (Magunga and Kilimanjaro) where scaled ITN coverage may also have resulted in a difference between "historical" estimations of *Pf*PR_2–10 _and the current values used to match the clinical surveillance period [48].

More importantly the differences in peak age of hospitalized malaria disease presentation and transmission intensity have implications for the design of suites of prevention strategies planned for the control of malaria in Africa. The benefit of targeted intervention in the use of bed nets for malaria control as currently recommended by the WHO is based on the reasoning that targeting bed nets to the highest risk groups; infants and pregnant women, achieves the highest public health impact. Following the same reasoning it is clear that the likely public health impact of intermittent presumptive treatment of infants (IPTi) coincidental with vaccine schedules [49], is likely to be greatest in areas of the transmission axis where the disease burden in concentrated in infancy (Figure 3a). As the estimate of *Pf*PR_2–10 _declines IPTi must adapt to include increasingly older age risk groups [50] until one considers adoption of IPT in school-aged children [51] (Figure 3b). It seems entirely plausible that with adequate scaling of ITN coverage in most areas of Africa where *Pf*PR_2–10 _starts at values <40% a dramatic reduction in transmission intensity is likely within 3–5 years [52], as this happens the age-patterns of severe malaria presenting to hospital will change and increasingly become less dominated by infants, making the adaptation of the IPTi rationale an immediate priority. Following the same reasoning, outpatient screening tools such as the WHO recommended IMCI guidelines currently in use across many African countries will need to be modified to accommodate expected changes in disease presentation. The current dogma is that across Africa hospitalized malaria is a young paediatric problem, however recent assemblies of *Pf*PR_2–10 _information from across the continent [34], suggest that the predominant transmission pattern is one approximating to areas closer to the left hand side of the X-axis of Figures 3a and 3b. Areas of exceptionally high transmission are likely to be less common than previously thought and yet are often the choices of location for most clinical studies of hospitalized malaria in childhood.

In this study series, cerebral malaria appeared to be a more common presentation among children hospitalized with malaria from lower intensity transmission settings compared to areas of high transmission (Figure 4a). This observation has been made in other between site comparative studies [2,3,13,17,24,53]. The proportion of malaria admissions regarded as having a BCS ≤ 2 varied considerably under a wide range of transmission conditions from *Pf*PR_2–10 _1% to 33%, however, and may also reflect the difficulties in measuring cerebral malaria in very young children [54]. The proportion of children presenting with severe malaria anaemia showed little variation across the range of transmission conditions from 1–80% *Pf*PR_2–10 _(Figure 4b) with the exception of the highest recorded proportion of anemic children (50%) from Nyamawala/Michenga villages where *Pf*PR_2–10 _was 87%. The epidemiology of severe malaria anaemia may be more complex than previously thought [12,55] and while is a common feature in young hospitalized infants remains a clinical predictor in older children [30]. A recent study looking at severe anaemia in children indicates that the occurrence of SMA is more likely to be multi-factorial than is CM and importantly is also more likely to be context specific relating to nutrition, prevalence of HIV and prevalence of other diseases which are associated with severe anaemia [56], thus complicating direct comparisons between sites.

The incidence of hospitalization has not been examined, largely because the precise calculation of the paediatric populations at risk was not possible across most of the sites studied after the 1990's. Therefore, no specific comments on the overall changing risks of hospitalization as *Pf*PR_2–10 _declines can be made. Nevertheless, it is interesting to note that the one long-term serial study of severe clinical malaria in Africa has investigated the rate of hospitalization during a time of major transmission reduction at Kilifi North [41]. O'Meara et al. (2008) showed that in this community, that began with a *Pf*PR_2–10 _of approximately 50% and declined to 1% over ten years, resulted in a ten-fold decline in the risks of hospitalization with malaria in childhood. The focus here has been on better descriptions, across more sites of the age and clinical presentation of hospitalized malaria in childhood likely to be observed with reductions in transmission intensity across Africa as prevention strategies go to scale over the next ten years. Perhaps not surprisingly these results confirm many other observations, using less rigorous inclusion criteria [11,23,25], that declining transmission intensity will result in fewer infants and proportionately more children of older age groups as representing the clinical burdens facing hospitals in Africa. It is less certain whether the case-mix of cerebral malaria and severe malaria anaemia will change coincidental with declining transmission intensity.

## Competing interests

The authors declare that they have no competing interests.

## Authors' contributions

EA assembled all the hospital data, restructured the data and wrote the manuscript; AAT, HR, RI and JAB were responsible for the assembly of hospital data from Yemen, Tanzania, Uganda and Kilifi, Kenya respectively and contributed to the final manuscript. RWS was responsible for the project and its overall scientific management, interpretation and preparation of the final manuscript.
